# Avian Influenza Virus Surveillance in Wild Birds in Georgia: 2009–2011

**DOI:** 10.1371/journal.pone.0058534

**Published:** 2013-03-13

**Authors:** Nicola S. Lewis, Zurab Javakhishvili, Colin A. Russell, Ann Machablishvili, Pascal Lexmond, Josanne H. Verhagen, Oanh Vuong, Tinatin Onashvili, Marina Donduashvili, Derek J. Smith, Ron A. M. Fouchier

**Affiliations:** 1 Department of Zoology, University of Cambridge, Cambridge, United Kingdom; 2 Department of Virology and National Influenza Center, Erasmus Medical Center, Rotterdam, The Netherlands; 3 Fogarty International Center, National Institutes of Health, Bethesda, Maryland, United States of America; 4 Institute of Ecology, Ilia State University, Tbilisi, Georgia; 5 Ivane Javakhishvili Tbilisi State University, Tbilisi, Georgia; 6 Influenza Section, National Centre for Disease Control, Tbilisi, Georgia; 7 Laboratory of the Ministry of Agriculture, Tbilisi, Georgia; University of Georgia, United States of America

## Abstract

The Caucasus, at the border of Europe and Asia, is important for migration and over-wintering of wild waterbirds. Three flyways, the Central Asian, East Africa-West Asia, and Mediterranean/Black Sea flyways, converge in the Caucasus region. Thus, the Caucasus region might act as a migratory bridge for influenza virus transmission when birds aggregate in high concentrations in the post-breeding, migrating and overwintering periods. Since August 2009, we have established a surveillance network for influenza viruses in wild birds, using five sample areas geographically spread throughout suitable habitats in both eastern and western Georgia. We took paired tracheal and cloacal swabs and fresh feces samples. We collected 8343 swabs from 76 species belonging to 17 families in 11 orders of birds, of which 84 were real-time RT-PCR positive for avian influenza virus (AIV). No highly pathogenic AIV (HPAIV) H5 or H7 viruses were detected. The overall AIV prevalence was 1.6%. We observed peak prevalence in large gulls during the autumn migration (5.3–9.8%), but peak prevalence in Black-headed Gulls in spring (4.2–13%). In ducks, we observed increased AIV prevalence during the autumn post-moult aggregations and migration stop-over period (6.3%) but at lower levels to those observed in other more northerly post-moult areas in Eurasia. We observed another prevalence peak in the overwintering period (0.14–5.9%). Serological and virological monitoring of a breeding colony of Armenian Gulls showed that adult birds were seropositive on arrival at the breeding colony, but juveniles remained serologically and virologically negative for AIV throughout their time on the breeding grounds, in contrast to gull AIV data from other geographic regions. We show that close phylogenetic relatives of viruses isolated in Georgia are sourced from a wide geographic area throughout Western and Central Eurasia, and from areas that are represented by multiple different flyways, likely linking different host sub-populations.

## Introduction

Aquatic birds are the natural reservoir for all avian influenza A viruses (AIVs), and are subtyped according to 16 haemagglutinin (HA) subtypes and 9 neuraminidase (NA) subtypes [1], [2]. Most AIVs are of low pathogenicity and cause mild or subclinical infections in aquatic birds. Low pathogenic avian influenza (LPAI) viruses have been isolated from over 136 species of wild birds and are most commonly isolated from *Anseriformes* and *Charadriiformes* [3]. Despite widespread surveillance, there remain substantial unanswered questions about the spatial, temporal and ecological role of the host populations in defining the genetic structure of AIVs.

Since the emergence and westward spread of HPAI H5N1 from SE-Asia, one of the outstanding questions is the role wild birds, particularly long distance migrants, might play in the dissemination of AIV from SE-Asia to other geographic regions [4], [5]. The Caucasus region is crossed by thousands of migratory birds annually, and Georgia is located at the intersection of three wild bird migratory flyways – the Central Asian, East Africa-West Asia and Mediterranean/Black Sea. Additionally, the wetland habitats within Georgia are used as a migratory stop-over and over-wintering area for tens of thousands of ducks, and for breeding, migration stop-over and over-wintering for hundreds of thousands of gulls offering potential for AIV transmission among bird populations originating in different geographic areas.

AIV surveillance work on *Charadriiformes*, particularly waders and gull species has mainly been carried out in North America, north western Europe and Russia [6], [7], [8], [9], [10], [11], [12]. Gulls have been shown to harbor a variety of influenza subtypes, including H13 and H16, which almost exclusively occur in gulls and terns [1], [13]. High AIV prevalence during migration stopover has been observed, notably in waders, particularly the Ruddy turnstone, in Delaware Bay, on the East coast of the United States [14], [15]. What is not known is the role that *Charadriiformes* might play in AIV virus ecology and in the potential for AIV dissemination outside this wader species AIV hotspot in Delaware Bay, and in species and geographic regions outside North America.

Here we report the findings of a longitudinal study set up in the Republic of Georgia in 2009 to investigate the ecology and evolution of AIV in wild birds in the Caucasus. We test the hypothesis that Georgia acts as a hub for the transmission of AIV due to frequent mixing events among birds originating from different geographic areas. In addition, using Armenian Gulls (*Larus armenicus*) as a model species, we longitudinally track the antibody profile of this host population and the point at which any AIV infection might occur, to investigate the ecology of AIV in these Eurasian gulls.

## Materials and Methods

### The study area

**Figure 1:**
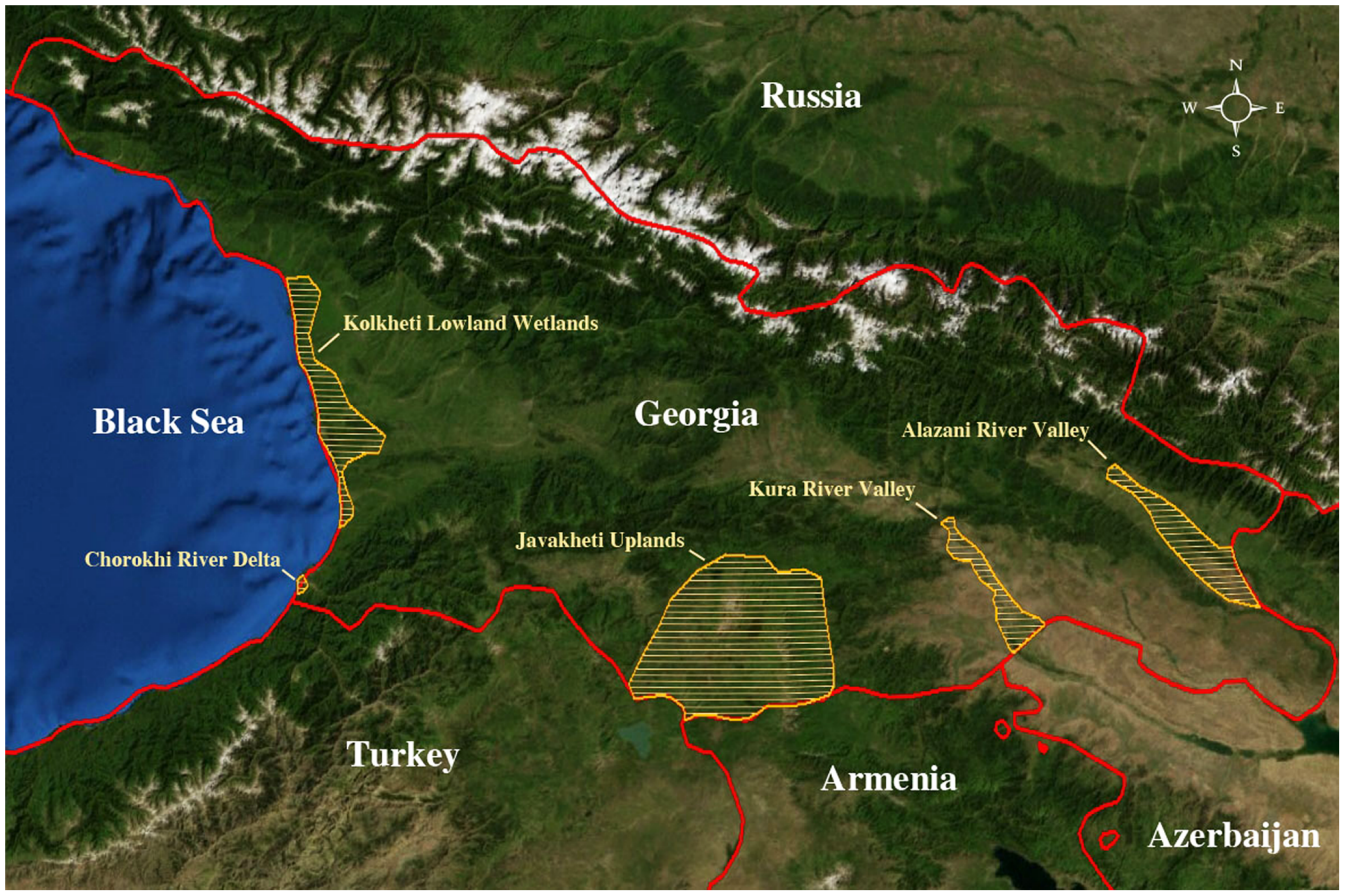
Map of Georgia. The study areas in Georgia are shown in yellow hatch, the country boundaries in red, and the main geographic features as an altitude relief.

Georgia is a Eurasian country in the Caucasus region, bordered by the Black Sea, Russia to the north, Azerbaijan to the east and by Turkey and Armenia to the south. It covers 69700 km^2^ with a population of approximately 4.7 million people. It is a country of geographic extremes ranging from humid subtropical and high mountain to semiarid and arid landscape types [16]. The primary wetlands include the Ramsar Wetlands in the Kolkheti Lowland Wetlands [17] the Javakheti Uplands, and the Kura River and Alasani River Valleys and tributaries which run from Turkey through Georgia and through Azerbaijan to enter the Caspian Sea. Sample sites were selected in collaboration with local ecologists and ornithologists to include all major wetland areas in Georgia (Figure 1).

### Capture and sampling methods and sample collection

Throughout the study period we targeted our surveillance towards *Anatidae* and *Charadriiformes* but also sampled other avian species commonly occurring in the Georgian wetland ecosystem (See Table S1).

We used several methods to catch birds depending on the species and location, including mist nets, spring traps and manual capture using hand-held nets, lamping and sampling hunted birds. Fresh fecal samples constituted approximately 70% of the samples taken from gulls but all after positive species identification. To do this we first observed the flock and ascertained whether it was made up of a single species. If so, we then flushed the birds and took fresh feces samples immediately from the area the birds had just occupied. To sample live-caught or hunted birds, a sterile plain cotton swab was inserted into the trachea or oropharynx (in smaller bird species), or approximately 5 mm into the cloaca of the bird and then gently turned to moisten the swab. All swabs were then inserted into viral transport storage media (Hanks balanced salt solution containing 10% glycerol, 200 U/ml penicillin, 200 mg/ml streptomycin, 100 U/ml polymixin B sulfate and 250 mg/ml gentamycin) and the shaft of the swab broken just above the cotton tip. Swabs were stored at −70°C no more than 6 hours after collection and were chilled at 1–4°C on ice or in a portable refrigerator in the interim. An in-depth discussion of potential sampling bias introduced by trapping method, which might influence prevalence or detection success of AIV is included in the supplementary online material (See Text S1).

### Sample timing

Sampling was carried out throughout the year. However, the seasonal fluctuation in bird density was affected by the natural ecology of the host.

#### Ducks

The number of breeding ducks is unknown but the main breeding area for ducks in Georgia is in the Javakheti Upland sampling area. In August, September and October these breeding populations concentrate on shallow lakes with vegetation cover for post-breeding moulting and are augmented by migrant ducks which appear to prefer the upland lakes as a stop-over and moult site over the Black Sea Coast wetland areas. The largest proportion of duck sampling occurred during the overwintering period on the Black Sea Coast where average mid-winter counts of ducks on the Kolkheti Lowland Wetlands is approximately 50,000 birds.

#### Quail

Tens of thousands of Common Quail (*Coturnix Coturnix*) migrate through the Alasani River Valley sampling area in September from their breeding sites in the Eurasian Steppe to their overwintering sites in the Middle East and East Africa.

#### Gulls

Six species - Armenian Gull (*Larus Armenicus*), Black-headed Gull (*Chroicocephalus ridibundus*), Yellow-legged Gull (*Larus michaellis*), Caspian Gull (*Larus Cachinnans*), Lesser Black-backed Gull (*Larus fuscus*), and Mediterranean Gull (*Larus melanocephalus*) were sampled in the overwintering period in the Kolkheti Lowland Wetlands, Chorokhi River Delta and the Kura River Valley. In addition we intensively sampled a large breeding colony of Armenian Gulls (*Larus Armenicus*) in the Javakheti Uplands through May–October. Other gull species were sampled infrequently (see Table S1).

### Diagnostics and virus isolation

RNA was isolated using a MagnaPure LC system with the MagnaPure LC Total nucleic acid isolation kit (Roche Diagnostics, Almere, Netherlands) and influenza A virus was detected using an in-house real-time RT-PCR (RRT-PCR) assay targeting the matrix gene (14). Amplification and detection of the matrix gene segment (M) was performed on an ABI7500 Real-Time PCR System (Applied Biosystems, CA, USA) with the TaqMan EZ RT-PCR Core Reagents kit (Applied Biosystems, CA, USA) using 20 µl of eluate in an end volume of 50 µl. Pooled individual samples were prepared and processed in parallel with several negative (three negative controls per 32 samples) and 2 positive control samples per 32 samples: 1 H5 and 1 H7 AIV isolate. Upon identification of influenza A virus positive pools, the RNA isolation and RRT-PCR procedures were repeated for the individual samples within each positive pool (again processed in parallel with three negative controls and two positive controls per 32 samples). All matrix gene segment real-time RT-PCR (M RRT-PCR) positive samples were subsequently used for virus isolation. RNA isolation and RRT-PCR were performed by the diagnostic facility of the Erasmus MC Department of Virology.

All M RRT-PCR samples were immediately tested by RRT-PCR that was specifically designed to detect either the H5 or the H7 HA gene subtypes. The HA gene of H5 and H7 positive samples were re-amplified by reverse transcriptase PCR and sequences to discriminate LPAI from HPAI viruses. If samples were found to be H5 or H7 positive in RRT-PCR and after confirmation that no multi-basic cleavage site was present in the HA gene of the relevant strain, 200 ul of the original specimen was inoculated for virus isolation in 11-day old embryonated chicken eggs. All other influenza virus positive samples that were found to be solely Matrix-positive (i.e. not H5 of H7 positive) were inoculated in embryonated chicken eggs directly for virus propagation.

Virus isolates were identified in a haemagglutination assay with turkey red blood cells. Subsequently, the HA of the virus was characterized using a panel of 24 hyperimmune rabbit antisera specific for each of the 16 HA subtypes isolated from birds (for some subtypes, more than 1 antiserum was used). The NA subtype was characterized by RT-PCR and sequenced using primers specific for the non-coding regions of NA (4). 1000 of the 8343 analyzed swabs were processed through the Laboratory of the Ministry of Agriculture of Georgia. In this case, RNA was isolated using a Qiagen RNA extraction minikit and influenza A virus was detected using a RRT-PCR assay targeting the matrix gene [18] using a Qiagen One-Step RT-PCR Kit and the Roche Lightcycler 2 (Roche, IN, USA).

### Sequencing

PCR products were purified from agarose gels using the Qiagen Qiaquick Gel Extraction kit and sequenced. Sequencing of HA and NA was performed using the Big Dye terminator sequencing kit v3.1 (GE Healthcare Life Sciences, Diegen, Belgium) and a 3130x1 genetic analyzer (Applied Biosystems, CA, USA), according to the manufacturer's instructions.

### Maximum likelihood phylogenetic analyses

We acquired HA and NA sequences from wild bird LPAI viruses from the NIAID IRD online [19] on 23/03/2011. We focused on H1-H12, which show no previously observed species-specific infection bias but also included H13 (plus the NA associated with each H13 strain) when H13 viruses were identified in Georgia. To this dataset we added the HA and NA LPAI sequences from Georgian wild birds collected as part of this study. (GenBank accession numbers KC190165-KC190184 and KC541676-KC541700), Sequences were aligned using MUSCLE. We inferred a maximum likelihood (ML) phylogenetic tree for the HA1 and NA nucleotide sequences using PAUP* (version 4.0b10) [20] using GTR+I+Γ_4_ (the general time-reversible model with the proportion of invariant sites and the gamma distribution of among-site rate variation with four categories estimated from the empirical data) as determined by ModelTest [21]. Global optimization of the tree topology was performed by tree bisection-reconnection branch swapping. The robustness of individual nodes of the tree was assessed using a bootstrap resampling analysis (100 replicates, with topologies inferred using the neighbor-joining method under the GTR+I+Γ_4_ substitution model).

### Serology, collection, and testing

We sampled approximately 0.5–1 ml venous blood (depending on species) from either brachial or tarsal veins using aseptic technique and placed the blood in gel serum separator plain blood tubes (Greiner Bio-One, MiniCollect, 0.8 ml Z Serum Sep). Clotted blood samples were spun at 3000 g for 10 minutes to separate the serum from the cellular component, approximately 6–8 hours after collection. Serum samples were stored at −20 degrees C prior to serological analyses.

We tested for the presence of antibodies to nucleoprotein (NP) in individual serum samples using a commercial blocking enzyme-linked immunosorbent assay as per the manufacturers instructions, together with the supplied positive and negative controls (bELISA MultiS-Screen Avian Influenza Virus Antibody Test Kit, IDEXX Laboratories). All samples were run in duplicate. Serological positives were estimated by eye, as there was no plate reader available in Georgia. Because of the qualitative rather than quantitative read-out of the assay, only strong positives or strong negatives were considered in the following results section, which might underestimate the sero positive rates in gulls.

## Results

We collected 8,343 swabs from 76 species of birds, belonging to 17 families in 11 orders. Of these, 84 samples were M RRT-PCR positive (1%) (Table 1). No highly pathogenic avian influenza (HPAI) H5 or H7 viruses were detected from any of the samples analyzed during this study as assessed by standard sequence-based methods. Table 1 shows the number of samples taken from species groups, the number of birds sampled, the number of M RRT-PCR positive birds, the percentage M RRT-PCR positive and the 95% confidence interval associated with the prevalence estimate. Species sampled in each group are shown in Table S1. Of the M RRT-PCR positive samples, 66% were taken from gulls, 30% from dabbling ducks, 3% from rails and crakes, 1% from other waterbirds, and 1% from Common Quail.

**Table 1:** Number of samples taken from species groups, the number of birds sampled, the number of positive birds, the percentage positive and the 95% confidence interval associated with the prevalence estimate.

Species Group	No. of samples	No. of birds sampled	No. of AIV-positive birds	% birds positive	95% confidence interval
**Dabbling ducks**	2845	1418	25	1.76	0.09
**Diving ducks**	142	71	0	0	*
**Other ducks**	54	27	2	7.4	2.79
**Other waterbirds**	273	136	1	0.7	0.05
**Geese**	36	18	0	0	*
**Gulls**	2994	2545	53	2	0.08
**Galliformes**	796	398	1	0.25	0.007
**Rails and Crakes**	845	422	2	0.47	0.01
**Terns**	261	131	0	0	*
**Passerines**	65	33	0	0	*
**Raptors**	42	21	0	0	*
	8343	5220	84	1.62	0.020

The bird species included in each species group are shown in Table S1.

To avoid inaccurate reporting of prevalence estimates because total swab count might include two swabs from one bird (tracheal and cloacal), or only an environmental sample derived from fresh feces, we also report percentage prevalence by number of birds sampled. 1.69% of all ducks, and 2% of all gulls were M RRT-PCR positive for influenza viruses. Table 2 shows the prevalences (%) for each duck and gull species and for other waterbirds that were RRT-PCR positive for AIVs.

**Table 2:** Percentage prevalences for each duck, gull and other waterbird species, which were positive for AIV.

Species	Latin name	Number positive	Number sampled	% Prevalence
**Mallard**	Anas Playrhynchos	21	1185	1.7
**Northern Shoveler**	Anas clypeata	1	10	10
**Common Goldeneye**	Bucephala clangula	2	9	22
**Common Teal**	Anas crecca	1	126	0.79
**Northern Pintail**	Anas acuta	1	17	5.9
**Garganey**	Anas querquedula	1	75	1.3
**Gadwall**	Anas strepera	0	16	-
**Smew**	Mergellus albellus	0	2	-
**Eurasian Wigeon**	Anas Penelope	0	2	-
**Red-breasted Merganser**	Mergus serrator	0	5	-
**Ruddy Shelduck**	Tadorna ferruginea	0	10	-
**Common Shelduck**	Tadorna tadorna	0	5	-
**Tufted Duck**	Aythya fuligula	0	42	-
**Common Pochard**	Aythya farina	0	22	-
**Velvet Scoter**	Melanitta fusca	0	2	-
**Red-crested Pochard**	Netta rufina	0	3	-
**Greater Scaup**	Aythya marila	0	8	-
**Ferrugineous Duck**	Aythya nyroca	0	3	-
**Armenian Gull**	Larus armenicus	6	624	0.96
**Black-headed Gull**	Chroicocephalus ridibundus	36	526	6.8
**Yellow-legged Gull**	Larus michahellis	9	1328	0.67
**Caspian Gull**	Larus cachinnans	1	1	N/A
**Little Grebe**	Tachybaptus ruficollis	1	9	11
**Common Moorhen**	Gallinula chloropus	1	68	1.4
**Eurasian Coot**	Fulica atra	2	243	0.8

We next investigated whether it was necessary to take both tracheal and cloacal swabs to detect AIVs in wild birds. Previous experimental work has also shown that patterns of virus attachment of avian influenza viruses differs among even closely related avian species [22] so we also assessed whether the predominant route of AIV shedding might differ among ducks and gulls sampled in Georgia. We took paired tracheal and cloacal swabs from all ducks. Of these duck swabs, 87.5% (28/32) of the AIV-positives originated from cloacal swabs and 12.5% (4/32) from tracheal swabs.

In gulls, 74% (2192/2994) of all swabs were taken from fresh feces after pre-species identification, and 13% (398/2994) each from paired trachea and cloacal sampling. Of the AIV-positive sampled taken from gulls, 69% (37/53) were obtained from fresh feces, 274% (13/53) from cloacal swabs and only 6% (3/53) from tracheal swabs. No individual bird was sampled as AIV- positive from both tracheal and cloacal swabs

After excluding the gull fresh feces samples from the analyses as paired samples were not taken, we found a significant difference between the number of AIV-positive tracheal and the number of AIV-positive cloacal samples with disproportionately more cloacal than tracheal-positive samples (Chi-squared test for given probabilities: X-squared = 27.76, p-value = <<<0.05).

However there is no significant difference between the respiratory and cloacal shedding patterns between gulls and ducks (Pearson's chi-squared test with Yates continuity correction: X-squared = 0.02, p-value = 0.885). These results suggest that it is important to take both tracheal and cloacal samples if possible, because a) no bird was detected as M RRT-PCR positive through both tracheal and cloacal swabs and therefore AIV-positive birds might be missed, and b) although we observed no significant difference in the source of AIV-positive swabs we cannot exclude that predominant shedding patterns might differ among different subtypes, by host, and by amount of virus from different routes, so potentially affecting not only detection if one does not take both tracheal and cloacal swabs, but also virus isolation success.

Therefore we also considered whether the ability to isolate virus from gulls or ducks differed in terms of route or amount of virus excreted by a certain route, or whether particular subtypes were shed by a particular route. 49 fresh faeces, cloacal or tracheal samples were RRT-PCR MA-positive from gulls (CT-values: 15–39) (Table S2). 13 viruses were isolated from these RRT-PCR positive samples, all from fresh faeces or cloacal samples (CT-values of isolates: 18–32). Only 3 tracheal samples were RRT-PCR-positive (CT-values: 31–36) and no isolates were derived from tracheal samples. H11N1, H9N1, H9N3, H13N6 and H13N8 were isolated from gulls, from cloacal and fresh faeces samples. These results suggest that faeces were the predominant route of shedding as quantified by RRT-PCR, and that H9, H11 and H13 are shed effectively by this route in this host. The very limited number of tracheal swab RRT-PCR positive samples, the high CT-values and the lack of virus isolates suggest that this is the less important route of shedding in this study and that taking fresh faecal or cloacal swabs is imperative to detect AIV infection and potentially isolate the subtypes circulating (even uncharacterized) in gulls.

In ducks, 31 fresh faeces, cloacal or tracheal samples were RRT-PCR MA-positive (CT-values: 22–38) (Table S2). 11 viruses were isolated from these RRT-PCR positive samples, from fresh faeces, tracheal and cloacal swabs (CT-values of isolates: 22–32). Of the 5 isolates from tracheal swabs, we characterised an H3N8, an H7/H10N1 mixed infection and H7N7 virus. We subtyped H1N1, H2N3, H3N8, H4N2, H7N3, and H10N4 and from the 6 isolates derived from cloacal swabs. Although the number of samples was too small to statistically test, the CT-values for tracheal or faecal swab-positive H7 viruses were similar suggesting no predilection of either shedding route. No other subtype was isolated from both tracheal and cloacal swabs but further data are needed to test for shedding patterns by subtype and for subtype isolation from particular species of duck.

**Figure 2:**
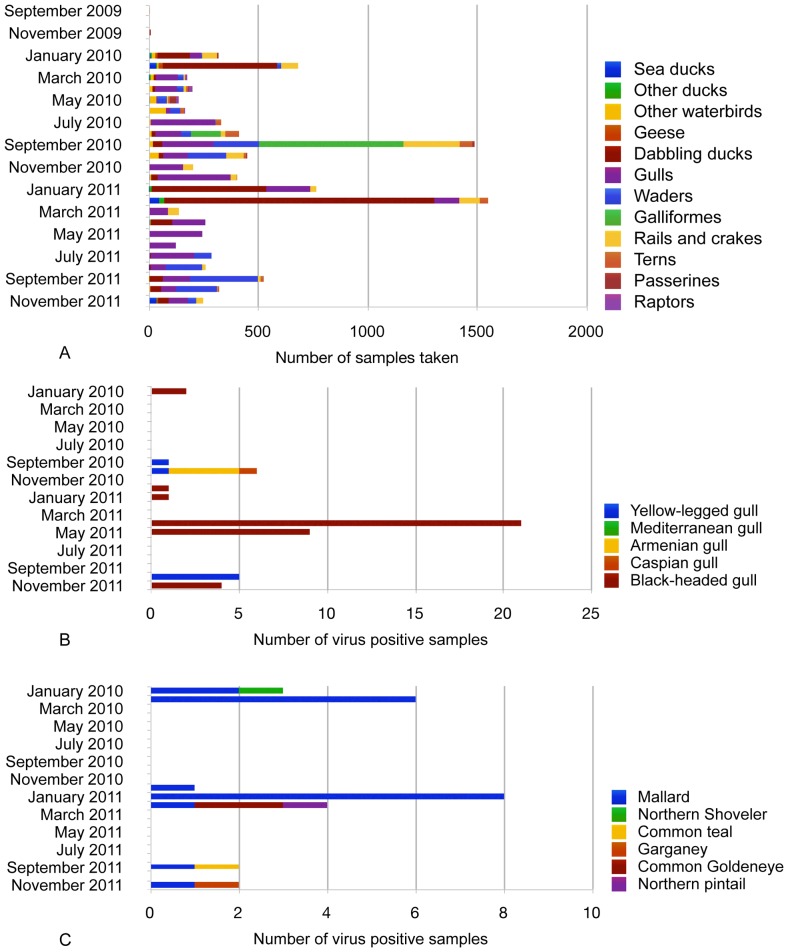
A–C. Number of samples taken during the study period. The number of samples by species (A), number of virus positive gull samples taken by species (B) and the number of virus positive duck samples taken by species (C) through January 2010–November 2011.

**Figure 3:**
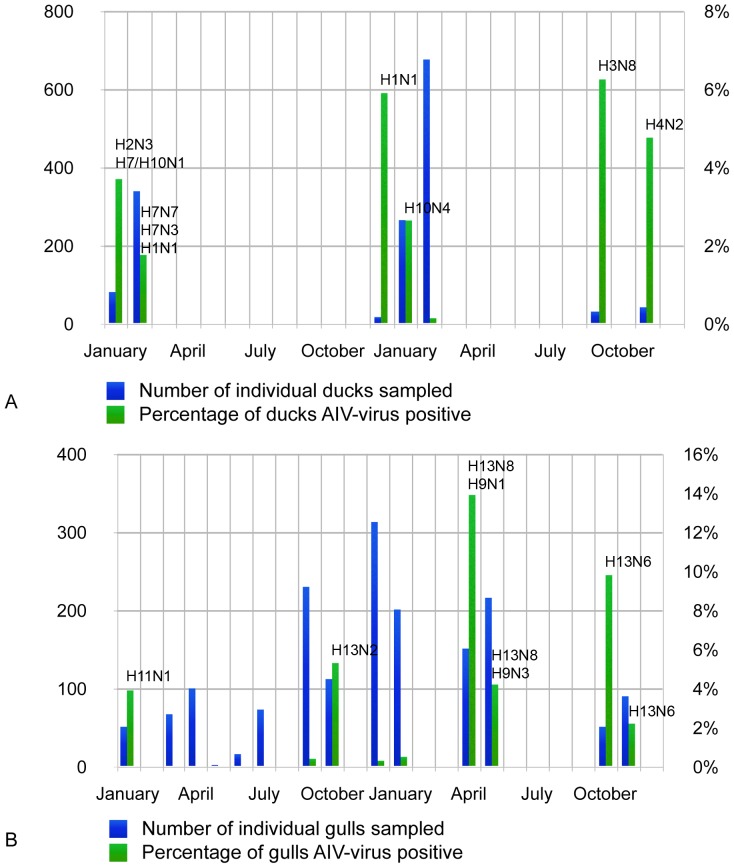
A–B. Longitudinal surveillance effort and AIV prevalence and subtype data in the two main species groups. Number of individual ducks (A) and gulls (B) sampled, the percentage of AIV positive birds and the subtype isolated during the study period January 2010–November 2011.

Figure 2 shows the number of samples from all species groups through the time period (A), the times of year when individual gull (B) and duck (C) species were M RRT-PCR positive. Peak prevalences in large gulls (Armenian Gull, Caspian Gull and Yellow-legged Gull) were seen during the autumn migration periods (5.3–9.8%), whereas in Black-headed Gulls, viruses were detected in a two-week window in April and May 2011 (4.2–13.9%) (Figure 3). These infections occurred in over-wintering gulls prior to their departure back to the breeding areas outside Georgia, but whilst other species were using the sample areas for spring migration-stopover. In ducks, increased AIV prevalence was associated with the autumn migratory period in the upland lakes (6.3%) and in the overwintering period in the Kolkheti wetlands (average 3.15% (range 0.14–5.9%) (Figure 3).

Single M RRT-PCR positive samples were also obtained from a Common Quail during migration in the east of Georgia in September 2010, a Common Coot in January 2010 during overwintering on the Black Sea Coast, and a Moorhen and a Little Grebe in August and September 2010 respectively, during the migration period on the Black Sea Coast.

Through the two year study period, we isolated 23 viruses in total from 84 M RRT-PCR positive swabs, an isolation success rate of 27% with a mean CT-value of 26.28 (16.69–33.96: stdev 4.70) for isolated samples, versus a mean CT-value 29.36 (18.24–36.38: stdev 4.52) for samples which were M RRT-PCR positive but from which we were unable to isolate virus. We were generally successful isolating virus from M RRT-PCR positive samples with a CT-value of under 30 (Table S2). However we also note that we appeared somewhat more unsuccessful isolating virus from low CT-value samples if they were taken from gulls rather than ducks. This might indicate that some influenza A viruses in gulls do not optimally replicate to high titres in our current culture and isolation system.

We detected a large subtype diversity of LPAI viruses: with H1N1, H2N3, H3N8, H4N2, H7N3, H7N7, H10N1 and H10N4 isolated from ducks, an H6N2 isolate from a Common Coot, H11N1, H13N6, H13N8, H9N1 and H9N3 isolates from Black-headed Gulls, H9N3 from Mediterranean gulls, and H13N2 and H13N6 from Yellow-legged Gulls. One Mallard sampled in 2010 had a mixed H7 and H10 infection.

To investigate the role that *Charadriiformes* might play in the ecology of AIVs in Eurasia, we longitudinally studied a breeding colony of Armenian gulls. Madatapa Lake, in the Javakheti Uplands, hosts a breeding colony of approximately 3000 pairs of gulls. The sample site lies at an elevation of 2109 metres and is thus frozen from November–April. In May 2011 we caught and serologically tested adult birds for influenza virus antibodies, of which 56% were positive (9 of 16). 53 tracheal and cloacal swabs taken at the same sampling were all virologically negative. We predicted that the chicks would have maternally-derived antibodies (MDA) for a period and indeed, from our individual chick age data (based on the degree of growth of juvenile flight plumage) 50% of chicks caught in the first month were sero-positive, with sero-positivity being recorded in the younger chicks, likely related to MDA. After one month from when the first chicks hatched, we did not sample a) any chicks with downy juvenile plumage, or b) record any sero-positive birds, likely because any MDA had waned and we were sampling older birds. However, we continued to use serological testing as any evidence of seroconversion would allow us to target which swabs taken for virological testing to prioritise through M RRT-PCR diagnostic screening to detect what we predicted would be a peak on infection in juveniles around the point of fledge. Of 328 swabs tested and 143 sera tested subsequently, none were virologically or serologically positive for AIV or antibodies. At the end of September the gulls left the breeding area. However, in October and November 2011, we found M RRT-PCR positive Armenian gulls in both the Kura River Valley sample area and the Kolkheti Lowland wetlands. Although not definitively part of the same population of breeding gulls, it is interesting to note that prior to the breeding colony moving out from Madatapa lake there were no observations of *Larus Armenicus* in either of the two putative post-breeding and overwintering areas of the Kura River basin and the Kolkheti wetlands.

We sequenced the HA and NA gene segments for 23 virus isolates to characterize the genetic evolution of LPAI viruses in Georgia relative to other circulating AIVs in Eurasia. In ducks, the HA and NA sequences were overall phylogenetically similar to others in Eurasia, but either a) grouped within-clade with solely Georgian sequences or b) within clades with relatively long branch lengths to sequences derived from other geographic areas, or c) phylogenetically characterized as a unique clade, suggesting that AIV sequence data density is not yet great enough to robustly test hypotheses about the spatial and temporal patterns in the evolution of Eurasian AIVs in ducks but that this Georgian dataset has captured previously unobserved genetic diversity.

In gulls, we observed that Georgian HA and NA sequences always grouped within clades containing only gull-derived isolates. Furthermore, these clades often only contained Georgian sequences. For example, H13 viruses formed two clades containing only Georgian isolates and a third clade where the closest relative was a virus isolated from a gull in Norway. To date, H13 and H16 have been regarded as predominantly species-specific to gulls. Here, however we observed that both H9 and H11 viruses derived from gulls formed phylogenetic clades distinct from circulating duck H9 and H11 viruses. For example, the H11N1 virus isolated from a Black-headed Gull in 2010 was phylogenetically distinct from other Eurasian H11 viruses, with the exception of a virus isolated from a gull in Kazakhstan. BLAST analyses in NCBI against published HA sequences showed a 97% similarity with this Kazakhstan virus and only 93% similarity with viruses isolated from shorebirds and ducks. We also compared 44 H11 Eurasian virus isolates including 3 gull isolates, 2 currently unpublished, which also showed close similarity among the gull isolates and dissimilarity from the Eurasian duck isolates. Whilst not conclusive owing to the extremely limited samples available, the H11 viruses from Kazakhstan and Georgia might represent a ‘gull’ lineage of H11 viruses similar to the species-specific lineages seen in H13 and H16. Likewise the H9 viruses might contain a lineage, which is species-specific to gulls. We note that we observed no overlap in the subtypes in the two orders of birds (*anseriformes* and *charadriiformes*). These substantial phylogenetic differences are notable and highlight the need for improved surveillance not only in ducks, but also in gulls and terns to investigate the ecology and evolution in these species groups.

**Figure 4:**
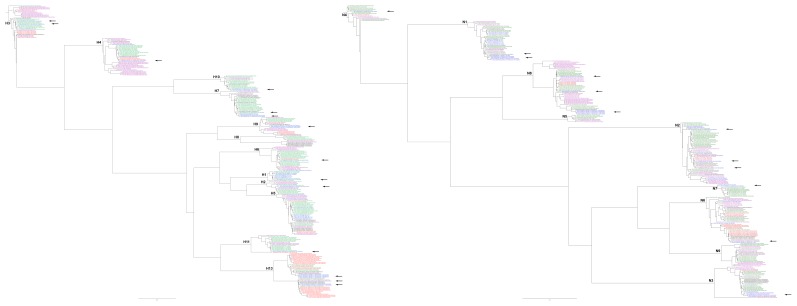
Maximum likelihood phylogenetic trees based on HA (left) and NA (right) nucleotide sequences of low pathogenic avian influenza A viruses isolated from wild birds between 1956–2011. The isolate names in the tree are colored according to migratory flyway: East Atlantic (green), Black Sea-Mediterranean (blue), East Africa-West Asia (red), Central Asia (black), East Asia Australian (purple). The isolates from Georgia are marked with a black asterisk and the subtype indicated on the panel.

**Figure 5:**
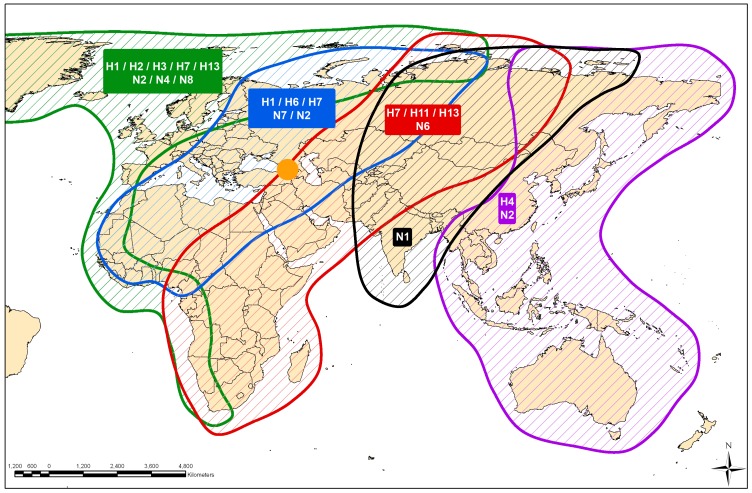
Flyway map of Eurasia showing the location and subtype of the closest phylogenetic relative to each Georgian isolate. Georgia is shown as an orange circle and the subtype icons are colored according to the flyway in which the place of isolation lies. The flyway colors are: East Atlantic (green), Black Sea-Mediterranean (blue), East Africa-West Asia (red), Central Asia (black), East Asia Australian (purple).

If Georgia acts as a hub for the transmission of viruses from one geographic area to another when birds aggregate for important stages of their life-cycle, one would predict firstly that the closest relatives to viruses isolated in Georgia, would be isolated from a wide geographical area throughout Eurasia and Africa. One would also predict that other areas where birds might aggregate but are not a hub for transmission might only yield closest relatives from a more limited geographic region. To test whether Georgia is a hub for transmission exhibiting both high virus diversity and high population mixing, we first constructed ML phylogenetic trees (Figure 4). We observed that the closest relatives to Georgian viruses were geographically spread throughout Central and South Asia, and Western Europe, and from both more northerly and southerly latitudes. The predominance of closest relatives from more western areas of Europe might reflect the greater level of wild bird surveillance that is carried out in this region. Flyway maps have been constructed primarily from bird ring data [5] and we can use such maps as a surrogate for the linkage among host populations within Eurasia and Africa. Integrating the phylogenetic and flyway data, we see that birds originating from different flyways likely mixed in Georgia as the origin of the closest relatives to the viruses they carried to Georgia were from the Black Sea-Mediterranean, the East Atlantic, the Central Asian and the East Asian/Australasian flyways (Figure 5). Although sufficient data are not yet available for more formal hypothesis testing, we also used the phylogenetic results here to test how relatively well-connected Georgia might be to bird populations in other geographic regions compared with countries to the east and west, both to inform important sites in which to carry out targeted surveillance to capture virus diversity, and to further test our hypotheses of the ecology and evolution of AIVs in the natural host. We measured the relative frequency with which viruses from a particular country are found in a phylogenetic clade together with other viruses from Europe, Central Asia or East Asia (Figure S1). We found that isolates from Sweden are always within European clades, viruses from the Netherlands nearly always, and viruses from Georgia nearly equally likely to be closely related to viruses from Europe, Central Asia or East Asia. Conversely, viruses from Mongolia and Russia were mostly closely related to viruses from East and Central Asia, and over 50% of viruses from China were mostly closely related to viruses from East Asia with a smaller proportion related to viruses from Europe and Central Asia. However, to fully test the characteristics required of an area to be considered key to the ecology of AIVs requires data from areas representing the full spectrum of host population mixing and a greater understanding of the host population ecology.

## Discussion

Ecological factors are likely to play a key role in the timing of LPAI virus infections observed in Georgia. Virus detection by M RRT-PCR from wild birds tended to coincide with migration or overwintering, particularly on the Black Sea coast and eastern sample sites. Interestingly the upland sample sites did not yield an M RRT-PCR-positive swab from *Charadriiformes* during the study period, despite intensive sampling of the breeding gull population, a high density of naive juvenile gulls, and detection of AIV in ducks, which were using the sample site during migration and post-breeding moult.

To account for these observations, one hypothesis is that three factors might be important in the ecology of gulls and AIVs in Georgia and the interplay among these factors affects the risk of AIV infection. At the start of the breeding season a) the population density of gulls is relatively high, b) susceptibility is relatively low because the population is predominantly adult (we found a seroprevalence rate of 50% in adult birds on the breeding grounds prior to hatch) and c) the probability of introduction of virus is likely small as the population is single species and colony-based. After hatch, and waning of maternally-derived antibodies after approximately 1 month of age, the population density is still high but population susceptibility also is now increased owing to the naive juveniles, but the probability of introduction of virus is still low. In our study, gulls did not become infected with AIVs until they moved off the breeding grounds to the overwintering grounds in September and October. Here the population density is still relatively high, susceptibility is high as birds arrive sero-negative from the breeding grounds, but now we hypothesize that the probability of introduction of virus is also high, whether as a result of increased mixing of gulls from different breeding colonies and areas, or contact with virus-infected species not present in the breeding areas. We also observed that the viruses isolated from gulls were H13, H9 or H11 subtypes, with H9 and H11, as well as H13, potentially from ‘gull’ lineages rather than phylogenetically like AIVs isolated from ducks. Thus, putatively the introduction of AIVs into gulls in Georgia is more influenced by the dynamics of infection within gulls similar to the dynamics of H13 and H16 AIV infections among colonies seen in other geographic regions (personal communication, Josanne Verhagen, Erasmus Medical Centre) rather than by the arrival of ducks for migration stop-over and post-breeding moult.

Our duck population sample is taken from predominantly migratory/post-moult ducks from September onwards in the Javakheti Uplands and overwintering ducks in the Kolkheti Lowland Wetlands. On Madatapa Lake in the Javakheti Uplands, we estimate 100–150 breeding pairs consisting of Northern Shoveler, Mallard, Garganey, Gadwall and Common Pochard. On a nearby lake there is a breeding population of Ruddy Shelduck. In late August and September local breeding populations of ducks aggregate to moult on the shallow lakes of Javakheti, their numbers augmented by the arrival of ducks from other breeding areas, using the site both to moult and as a migratory stop-over. This time is co-incident with the breeding gull population leaving the colony. Although previous data from northwest Europe and North America has suggested that peak prevalence in ducks occurs in the post-moult sites, where large numbers of naive juvenile birds mix in high density for the first time, prior to southward migration, here we find one peak in prevalence co-incident with post-breeding moult and autumn migration, followed by a period where there is low duck density and low or no prevalence, then another peak of similar prevalence when overwintering ducks arrive. Prevalence levels are not as high as seen in more northerly latitude post-moult study areas. Perhaps the majority of infections occur in the initial congregation sites and the lower levels we observed in Georgia are capturing the wave of virus dissemination as birds migrate and re-aggregate along the route, with birds from different geographic regions mixing, and differing by previous exposure history or other individual species-derived immunological factors. In both the gull and duck data, population density, susceptibility and probability of introduction through sub-population mixing appear to drive the dynamics of infection with LPAI viruses.

During the study period we only detected LPAI viruses and no HPAI viruses. This is despite the fact that during 2009–2012 HPAI outbreaks in wild birds were reported in other central Asian, African and Middle Eastern countries located within flyways that putatively also include Georgia (Wahid interface, OIE). The lack of HPAI viruses suggest that either the timing of such outbreaks relative to bird migration is critical to dissemination of HPAI viruses by wild birds, or that wild birds play a more limited role in the dissemination of HPAI viruses than has been thought. For example, if wild birds did play a substantial role in the dissemination of HPAI viruses, one would predict that outbreaks in other, more northerly Central Asian countries in March would likely pose little threat to Georgian bird populations if migration is south-north in that period. HPAI virus incursion threat to Georgia would likely be from countries where birds overwinter, further down the migratory route and the timing of active surveillance within Georgia could be targeted accordingly. Conversely, if wild birds play a more limited role in HPAI virus dissemination, then assessment of HPAI virus disease incursion risk to Georgia might focus on passive die-off reporting rather than active surveillance.

Understanding the ecology and evolution of AIVs in the natural host is key to understanding the role that wild birds might play in disseminating viruses among different geographic regions. From our data, geographic areas in which frequent migration events occur have the potential to influence virus genetic diversity.

We also observe that the closest geographic relatives to LPAI viruses isolated in Georgia are not solely isolated from countries to the west. Acquiring higher resolution data from Central and South Asia, particularly in terms of the LPAI viruses that circulate, is critical to establishing the relative inter-linking between different geographic regions and also the potential for AIV dissemination, particularly HPAI viruses, from east to west, mediated by wild birds.

To fully evaluate the factors that drive the evolution of AIVs we require a much greater understanding of the interplay between host species, environment, geography and time. Future work should include using the global AI sequence diversity to test for such interplay, particularly using methods that remove the confounding influence of some individual factors. Not only will this work give us key insight into the ecology and evolution of LPAI viruses in the natural host, but it will act as a basis for understanding some of the drivers that might be important when investigating the role that wild birds might play in HPAI virus spread, and the risk that this could pose to animal and public health

## Supporting Information

Table S1 **Bird species sampled for AIV surveillance in Georgia for each species group.**
(PDF)

Table S2 **Ecological, surveillance, laboratory diagnostic and virus subtype data (if available) for each swab tested AIV-positive by RRT-PCR in Georgia.**
(PDF)

Text S1 **An in-depth discussion of potential sampling bias introduced by trapping method, which might influence prevalence or detection success of AIV in Georgia.**
(PDF)

Figure S1 **Proportion of genetic clades containing viruses isolated from Europe (blue), Central Asia (green) and East Asia (gold) by country.**
(PDF)
