# Supplementary material for: Avian Influenza Virus Surveillance in Wild Birds in Georgia: 2009–2011
Source: PLoS One. 2013 Mar 13;8(3):e58534. doi: 10.1371/journal.pone.0058534 (PMC3596303; doi:10.1371/journal.pone.0058534)
Supplement: Table S1 — Bird species sampled for AIV surveillance in Georgia for each species group. (PDF) [file pone.0058534.s001.pdf]

Species Group										
**Dabbling ducks**	**Diving ducks**	**Other ducks**	**Other waterbirds**	**Geese**	**Gulls**	**Galliformes**	**Rails and Crakes**	**Terns**	**Passerines**	**Raptors**
Common Teal	Common Pochard	Common Goldeneye	Black-crowned Night Heron	Greylag Goose	Armenian Gull	Common Quail	Baillon’s Crake	Black Tern	Blackbird	Common Buzzard
Eurasian Wigeon	Ferrugineous duck	Common Shelduck	Cattle Egret	Lesser White-fronted Goose	Black-headed Gull		Common Coot	Caspian Tern	Common Kingfisher	Eurasian Sparrowhawk
Gadwall	Greater Scaup	Red-breasted Merganser	Demoiselle Crane	White-fronted Goose	Caspian Gull		Corn Crake	Common Tern	Common Starling	European Honey-buzzard
Garganey	Red-crested Pochard	Ruddy Shelduck	Glossy Ibis		Herring Gull		Little Crake	Little Tern	Common Woodpigeon	Levant Sparrowhawk
Mallard	Tufted Duck	Smew	Great Bittern		Lesser Black-backed Gull		Moorhen	Sandwich Tern	European Bee-eater	
Northern Pintail	Velvet Scoter		Great Cormorant		Little Gull		Purple Swamphen	Whiskered Tern	Grey Wagtail	
Northern Shoveler			Great Crested Grebe		Mediterranean Gull		Spotted Crake	White-winged Tern	Hooded Crow	
			Grey Heron		Mew Gull		Water Rail		Mistle Thrush	
			Little Bittern		Slender-billed Gull				Water Pipit	
			Little Egret		Yellow-legged Gull				White Wagtail	
			Little Grebe						Yellow Wagtail	
			Purple Heron							
			Pygmy Cormorant							
			Squacco Heron							
