# Supplementary material for: Avian Influenza Virus Surveillance in Wild Birds in Georgia: 2009–2011
Source: PLoS One. 2013 Mar 13;8(3):e58534. doi: 10.1371/journal.pone.0058534 (PMC3596303; doi:10.1371/journal.pone.0058534)
Supplement: Table S2 — Ecological, surveillance, laboratory diagnostic and virus subtype data (if available) for each swab tested AIV-positive by RRT-PCR in Georgia. (PDF) [file pone.0058534.s002.pdf]

Species (English)	Age (Adult, Juvenile, Undetermined)	Sex	Sampling site	Sample number	Date of sampling	Sample Type	MA-CT	H5	H7	H7-CT	Virus Isolation	E1	E2	Virus pathotype	HA subtype	NA subtype
**Black-headed Gull**	A	U	Poti	217-686	2010/01/21	cloacal swab	16.69	n	n		positive	0	1	LPAI	H11	N1
**Common Coot**	A	M	Natanebi	217-577	2010/01/22	tracheal or oropharyngeal swab	24.69	n	n		positive	0	1	LPAI	H6	N2
**Black-headed Gull**	A	U	Poti	217-716	2010/01/22	cloacal swab	28.36	n	n		negative	0		LPAI		
**Northern Shoveler**	A	F	Paliastomi lake	217-825	2010/01/30	cloacal swab	23.00	n	n		positive	1		LPAI	H2	N3
**Mallard**	A	F	Paliastomi lake	217-814	2010/01/30	tracheal or oropharyngeal swab	26.64	n	p	30.32	positive	1		LPAI	H10/H7	N1
**Mallard**	A	F	Paliastomi Lake	217-72	2010/01/31	cloacal swab	26.80	n	n		negative	0		LPAI		
**Mallard**	A	F	Hunting shop 1	217-968	2010/02/03	cloacal swab	28.59	n	n		negative	0		LPAI		
**Mallard**	A	F	Hunting shop 1	217-1050	2010/02/03	cloacal swab	29.86	n	p	32.85	positive	1		LPAI	H7	N3
**Mallard**	A	F	Hunting shop 1	217-1053	2010/02/03	cloacal swab	29.86	n	p	32.85	negative	0		LPAI		
**Mallard**	A	F	Hunting shop 1	217-974	2010/02/03	cloacal swab	29.97	n	n		negative	0		LPAI		
**Mallard**	A	F	Hunting shop 1	217-976	2010/02/03	cloacal swab	30.94	n	n		negative	0		LPAI		
**Mallard**	A	F	Hunting shop 1	217-1026	2010/02/03	tracheal or oropharyngeal swab	33.96	n	n		positive	1		LPAI	H7	N7
**Mallard**	A	M	Poti	217-1486	2010/02/18	cloacal swab	22.46	n	n		positive	1		LPAI	H1	N1
**Armenian Gull**	A	U	Poti	217-1660	2010/04/28	cloacal swab	27.45	n	n		negative	0		LPAI		
**Moorhen**	U	U	Natanebi	217-2665	2010/08/18	tracheal or oropharyngeal swab	33.40	n	n		negative	0		LPAI		
**Common Quail**	U	F	Telavi	217-3291	2010/08/30	cloacal swab	32.90	n	n		negative	0		LPAI		
**Little Grebe**	U	U	Natanebi	217-3774	2010/09/08	cloacal swab	26.87	n	n		negative	0		LPAI		
**Yellow-legged Gull**	U	U	Paliastomi Lake	217-4609	2010/09/29	cloacal swab	28.00	n	n		negative	0		LPAI		
**Armenian gull**	A	U	Paliastomi Lake	217-4660	2010/10/12	cloacal swab	18.24	n	n		negative	0		LPAI		
**Armenian gull**	A	U	Paliastomi Lake	217-4671	2010/10/12	cloacal swab	19.21	n	n		negative	0		LPAI		
**Armenian gull**	A	U	Paliastomi Lake	217-5250	2010/10/12	cloacal swab	22.59	n	n		negative	0		LPAI		
**Caspian Gull**	A	U	Paliastomi Lake	217-5197	2010/10/12	cloacal swab	28.10	n	n		negative	0		LPAI		
**Armenian gull**	A	U	Paliastomi Lake	217-4667	2010/10/12	cloacal swab	35.32	n	n		negative	0		LPAI		
**Yellow-legged Gull**	A	U	Chorokhi Delta	217-4691	2010/10/19	fresh feces	32.16	n	n		positive	1		LPAI	H13	N2
**Black-headed Gull**	A	U	Paliastomi Lake	217-5676	2010/12/07	cloacal swab	22.39	n	n		negative	0		LPAI		
**Mallard**	U	U	Paliastomi Lake	217-5542	2010/12/19	cloacal swab	25.44	n	n		negative	0		LPAI		
**Black-headed Gull**	A	U	Paliastomi Lake	217-6036	2011/01/05	cloacal swab	26.66	n	n		negative	0		LPAI		
**Black-headed Gull**	A	U	Paliastomi Lake	217-6042	2011/01/05	cloacal swab	26.85	n	n		negative	0		LPAI		
**Mallard**	U	F	Paliastomi Lake	217-5877	2011/01/23	cloacal swab	26.14	n	n		positive	1		LPAI	H1	N1
**Mallard**	U	F	Paliastomi Lake	217-5883	2011/01/23	cloacal swab	26.63	n	n		negative	0		LPAI		
**Mallard**	U	F	Paliastomi Lake	217-5939	2011/01/24	cloacal swab	30.52	n	n		negative	0		LPAI		
**Mallard**	U	F	Paliastomi Lake	217-5943	2011/01/24	cloacal swab	36.38	n	n		negative	0		LPAI		
**Mallard**	U	M	Paliastomi Lake	217-6142	2011/01/26	cloacal swab	27.40	n	n		negative	0		LPAI		
**Mallard**	U	M	Paliastomi Lake	217-6134	2011/01/26	cloacal swab	27.73	n	p	30.17	not performed			Not identifiable		
**Mallard**	U	F	Paliastomi Lake	217-6182	2011/01/31	cloacal swab	25.16	n	n		negative	0		LPAI		
**Mallard**	U	M	Paliastomi Lake	217-6322	2011/02/01	cloacal swab	27.43	n	n		negative	0		LPAI		
**Common Goldeneye**	U	F	Paliastomi Lake	217-7222	2011/02/08	cloacal swab	26.67	n	n		negative	0		LPAI		
**Common Goldeneye**	U	F	Paliastomi Lake	217-7220	2011/02/08	cloacal swab	32.19	n	n		negative	0		LPAI		
**Northern Pintail**	U	F	Paliastomi Lake	217-7322	2011/02/09	cloacal swab	22.50	n	n		negative	0		LPAI		
**Mallard**	U	F	Paliastomi Lake	217-7348	2011/02/09	cloacal swab	32.28	n	n		negative	0		LPAI		
**Mallard**	U	F	Paliastomi Lake	217-7502	2011/02/12	cloacal swab	28.04	n	n		positive	1		LPAI	H10	N4
**Black-headed Gull**	U	U	Paliastomi Lake	217-8458	2011/04/20	fresh feces	15.63	n	n		positive	1		LPAI	H13	N8
**Black-headed Gull**	U	U	Paliastomi Lake	217-8415	2011/04/20	fresh feces	16.38	n	n		positive	1		LPAI	H9	N1
**Black-headed Gull**	U	U	Paliastomi Lake	217-8388	2011/04/20	fresh feces	19.27	n	n		positive	1		LPAI	H13	N8
**Black-headed Gull**	U	U	Paliastomi Lake	217-8441	2011/04/20	fresh feces	22	n	n		positive	1		LPAI	H13	N8
**Black-headed Gull**	U	U	Paliastomi Lake	217-8436	2011/04/20	fresh feces	22.83	n	n		positive	1		LPAI	H13	N8
**Black-headed Gull**	U	U	Paliastomi Lake	217-8385	2011/04/20	fresh feces	29.11	n	n		negative	0		LPAI		
**Black-headed Gull**	U	U	Paliastomi Lake	217-8440	2011/04/20	fresh feces	34.64	n	n		negative	0		LPAI		
**Black-headed Gull**	U	U	Paliastomi Lake	217-8416	2011/04/20	fresh feces	35.94	n	n		negative	0		LPAI		
**Black-headed Gull**	U	U	Paliastomi Lake	217-8391	2011/04/20	fresh feces	36.18	n	n		negative	0		LPAI		
**Black-headed Gull**	U	U	Paliastomi Lake	217-8421	2011/04/20	fresh feces	36.73	n	n		negative	0		LPAI		
**Black-headed Gull**	U	U	Paliastomi Lake	217-8435	2011/04/20	fresh feces	36.88	n	n		negative	0		LPAI		
**Black-headed Gull**	U	U	Paliastomi Lake	217-8387	2011/04/20	fresh feces	36.91	n	n		negative	0		LPAI		
**Black-headed Gull**	U	U	Paliastomi Lake	217-8434	2011/04/20	fresh feces	37.01	n	n		negative	0		LPAI		
**Black-headed Gull**	U	U	Paliastomi Lake	217-8433	2011/04/20	fresh feces	37.52	n	n		negative	0		LPAI		
**Black-headed Gull**	U	U	Paliastomi Lake	217-8417	2011/04/20	fresh feces	38.12	n	n		negative	0		LPAI		
**Black-headed Gull**	U	U	Paliastomi Lake	217-8428	2011/04/20	fresh feces	38.26	n	n		negative	0		LPAI		
**Black-headed Gull**	U	U	Paliastomi Lake	217-8427	2011/04/20	fresh feces	38.4	n	n		negative	0		LPAI		
**Black-headed Gull**	U	U	Paliastomi Lake	217-8408	2011/04/20	fresh feces	38.54	n	n		negative	0		LPAI		
**Black-headed Gull**	U	U	Paliastomi Lake	217-8443	2011/04/20	fresh feces	39.38	n	n		negative	0		LPAI		
**Black-headed Gull**	U	U	Paliastomi Lake	217-8380	2011/05/02	fresh feces	20.52	n	n		positive	1		LPAI	H9	N3
**Black-headed Gull**	U	U	Paliastomi Lake	217-8444	2011/05/02	fresh feces	24.79	n	n		positive	1		LPAI	H9	N3
**Black-headed Gull**	U	U	Paliastomi Lake	217-8375	2011/05/02	fresh feces	34.01	n	n		negative	0		LPAI		
**Black-headed Gull**	U	U	Paliastomi Lake	217-8368	2011/05/02	fresh feces	35.82	n	n		negative	0		LPAI		
**Black-headed Gull**	U	U	Paliastomi Lake	217-8372	2011/05/02	fresh feces	36.37	n	n		negative	0		LPAI		
**Black-headed Gull**	U	U	Paliastomi Lake	217-8424	2011/05/02	fresh feces	37.42	n	n		negative	0		LPAI		
**Black-headed Gull**	U	U	Paliastomi Lake	217-8367	2011/05/02	fresh feces	38.33	n	n		negative	0		LPAI		
**Black-headed Gull**	U	U	Paliastomi Lake	217-8450	2011/05/02	fresh feces	38.40	n	n		negative	0		LPAI		
**Mediterranean Gull**	U	U	Paliastomi Lake	217-8989	2011/05/03	fresh feces	20.44	n	n		positive	1		LPAI	H9	N3
**Common Teal**	A	F	Madatapa	217-10028	2011/09/20	tracheal or oropharyngeal swab	22.78	n	n		positive	1		LPAI	H3	N8
**Mallard**	A	U	Madatapa	217-10049	2011/09/21	cloacal swab	25.66	n	n		positive	1		LPAI	H3	N8
**Yellow-legged Gull**	U	U	Paliastomi Lake	217-10377	2011/10/29	fresh feces	23.21	n	n		positive	1		LPAI	H13	N6
**Yellow-legged Gull**	J	U	Chorokhi Delta	217-10261	2011/10/30	cloacal swab	17.89	n	n		negative	0		LPAI		
**Yellow-legged Gull**	J	U	Chorokhi Delta	217-10260	2011/10/30	tracheal or oropharyngeal swab	31.06	n	n		negative	0		LPAI		
**Yellow-legged Gull**	A	U	Chorokhi Delta	217-10264	2011/10/30	tracheal or oropharyngeal swab	33.72	n	n		negative	0		LPAI		
**Yellow-legged Gull**	A	U	Chorokhi Delta	217-10262	2011/10/30	tracheal or oropharyngeal swab	36.7	n	n		negative	0		LPAI		
**Garganey**	A	F	Paliastomi Lake	217-10098	2011/11/08	cloacal swab	27.46	n	n		positive	1		LPAI	H4	N2
**Mallard**	A	M	Paliastomi Lake	217-10096	2011/11/08	cloacal swab	34.53	n	n		negative	0		LPAI		
**Yellow-legged Gull**	U	U	Tbilisi River	217-10184	2011/11/10	fresh feces	31.21	n	n		positive	1		LPAI	0	0
**Mallard**	A	M	Paliastomi Lake	217-9604	2011/11/14	tracheal or oropharyngeal swab	32.94	n	n		negative	0		LPAI		
**Mallard**	A	F	Paliastomi Lake	217-9603	2011/11/14	cloacal swab	36.68	n	n		negative	0		LPAI		
**Mallard**	A	M	Paliastomi Lake	217-9615	2011/11/14	cloacal swab	38.03	n	n		negative	0	0	LPAI		
**Black-headed Gull**	U	U	Paliastomi Lake	217-10221	2011/11/15	fresh feces	23.5	n	n		positive	1		LPAI	H13	N6
**Black-headed Gull**	U	U	Paliastomi Lake	217-10224	2011/11/15	fresh feces	39.36	n	n		negative	0		LPAI		
