# Supplementary material for: Avian Influenza Virus Surveillance in Wild Birds in Georgia: 2009–2011
Source: PLoS One. 2013 Mar 13;8(3):e58534. doi: 10.1371/journal.pone.0058534 (PMC3596303; doi:10.1371/journal.pone.0058534)
Supplement: Text S1 — An in-depth discussion of potential sampling bias introduced by trapping method, which might influence prevalence or detection success of AIV in Georgia. (PDF) [file pone.0058534.s003.pdf]

Trapping method and potential AIV prevalence or detection sampling bias

In this study, there were clearly dominant trapping methods used for different groups of birds.
Ducks:
The majority of duck samples were taken from hunted birds. Hunting in Georgia is not very focused on a particular species of duck and average hunter has difficulty in identifying the majority of species.
Hunters also hunt with several different methods e.g. driving a motor boat around the hunting site (open water on the sea, lake and river as well as up small channels in the marshes and flooded forest) and where they shoot flushed birds. Another method is to wait in hides near the shallow water with vegetation where ducks come for feeding. Hunters lure ducks with decoys and electric or mechanical calls. Hunters shoot any duck-like birds appearing within shooting distance of the hunter. Because of these differing methods and considering the large number of shot birds, we think it unlikely that sampling hunted birds introduces any sampling bias towards infected birds, thus having an influence of prevalence. However as the majority of duck samples result from a single sampling method we cannot compare with any statistical power, whether the trapping method definitively influences prevalence.
Gulls:
All nesting gulls were captured during the breeding period on the colony. All adults were captured on the nesting site on moonless nights using lamps and landing nets. We caught incubating birds as well as roosting birds to avoid gender bias.
Juveniles were captured during day again using a landing net.
We used suitable capture methods for particular bird groups and because these groups have fundamental ecological differences, we don't think it is appropriate to compare these two groups. However, post breeding/wintering gull samples were obtained using 2 major methods, fresh faeces and trapping. Using these data, we can test for capture method/prevalence if and when we obtain a statistically significant number of positive samples from both methods.
