# Supplementary material for: Avian Influenza Virus Surveillance in Wild Birds in Georgia: 2009–2011
Source: PLoS One. 2013 Mar 13;8(3):e58534. doi: 10.1371/journal.pone.0058534 (PMC3596303; doi:10.1371/journal.pone.0058534)

Proportion of genetic clades containing viruses isolated from Europe, Central Asia and East Asia by country

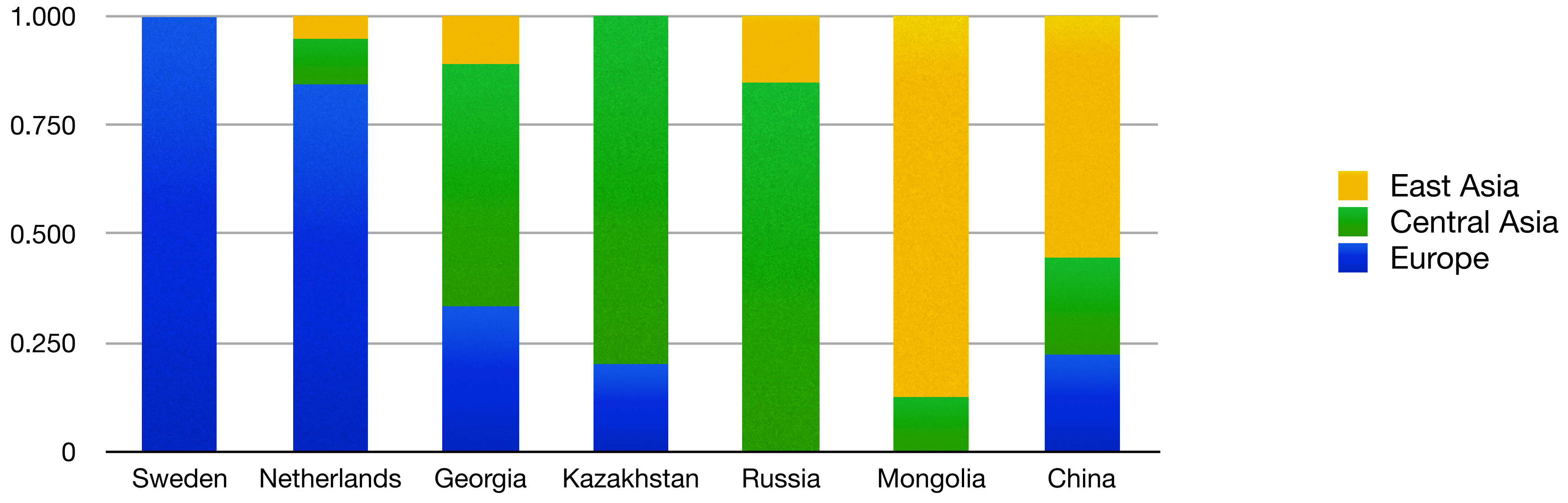

Supplement: Figure S1 — Proportion of genetic clades containing viruses isolated from Europe (blue), Central Asia (green) and East Asia (gold) by country. (PDF) [file pone.0058534.s004.pdf]
